# Negative Life Events and Psychological Distress Among Rural Chinese Older Adults with Hypertension: A Moderated Mediation Model of Self-Efficacy and Social Capital

**DOI:** 10.3390/healthcare14213638

**Published:** 2026-10-27

**Authors:** Yingjie Fu, Runqi Zhang, Shuo Zhang, Jiyao Sun, Xiaojie Sun, Yuanli Liu

**Affiliations:** 1 Affiliated Hospital of Shandong University of Traditional Chinese Medicine, Jinan 250014, China; 2 College of First Clinical Medicine, Shandong University of Traditional Chinese Medicine, Jinan 250014, China; 3 School of Health Management, Shandong University of Traditional Chinese Medicine, Jinan 250355, China; 4 School of Health Policy and Management, Chinese Academy of Medical Sciences and Peking Union Medical College, Beijing 100730, China; 5 Shandong Public Health Clinical Center, Jinan 250100, China; 6 Social Statistics, School of Social Sciences, The University of Manchester, HBS Building, Oxford Road, Manchester M13 9PL, UK; 7 Cathie Marsh Institute for Social Research (CMI), The University of Manchester, HBS Building, Oxford Road, Manchester M13 9PL, UK; 8 Centre for Health Management and Policy Research, School of Public Health, Cheeloo College of Medicine, Shandong University, No. 44 Wenhuaxi Road, Jinan 250012, China; 9 NHC Key Lab of Health Economics and Policy Research ( Shandong University), No. 44 Wenhuaxi Road, Jinan 250012, China

**Keywords:** life change events, psychological distress, hypertension, self-efficacy, social capital

## Abstract

**Highlights:**

**What are the main findings?**

- Negative life events were associated with greater psychological distress among rural older adults with hypertension, and self-efficacy accounted for part of this association.
- Higher social capital weakened both the direct association between negative life events and psychological distress and the indirect association through self-efficacy.

**What are the implications of the main findings?**

- Older adults with hypertension who experience negative life events, low self-efficacy, and limited social capital may represent a priority group for psychological assessment and support.
- Community-based strategies that strengthen self-efficacy and social resources may help support mental well-being among rural older adults with hypertension.

**Abstract:**

**Background:** Although negative life events have been linked to poorer mental health among older adults, how self-efficacy and social capital jointly relate to this association remains unclear among older adults with hypertension, who face additional demands of chronic disease management. We examined the mediating role of self-efficacy and the moderating role of social capital in the association between negative life events and psychological distress. **Methods:** This cross-sectional survey included 953 rural older adults with hypertension in Shandong, China. Negative life events in the preceding 12 months were self-reported. Psychological distress, self-efficacy, and social capital were measured using the Kessler Psychological Distress Scale (K10), General Self-Efficacy Scale (GSES), and Resource Generator Scale for China (RG-China), respectively. Hierarchical multiple linear regression and PROCESS Models 4 and 15 assessed mediation and moderated mediation using 5000 bootstrap resamples. **Results:** Negative life events were positively associated with psychological distress (β = 0.418). The indirect association through self-efficacy was 0.053 (95% CI: 0.035–0.073), accounting for 12.7% of the total association. Higher social capital was associated with a weaker positive association between negative life events and psychological distress (interaction β = −0.058) and a weaker negative association between self-efficacy and psychological distress (interaction β = 0.059). The index of moderated mediation was −0.012 (95% bootstrap CI: −0.023 to −0.001). **Conclusions:** Self-efficacy accounted for part of the association between negative life events and psychological distress, while social capital moderates both the direct and indirect associations. Social capital may be an important consideration in developing and evaluating future strategies to support psychological well-being and hypertension management among vulnerable older adults.

## 1. Introduction

Hypertension remains a major global public health challenge, affecting approximately one-third of adults worldwide and contributing substantially to cardiovascular disease and premature mortality [1,2]. In China, rapid population aging has further increased the challenges posed by hypertension, which remains highly prevalent and inadequately controlled [3,4,5]. Population-based studies have reported a higher prevalence of hypertension in rural than in urban communities, accompanied by lower rates of hypertension awareness, treatment, and control [6,7]. These disparities, compounded by population aging, pose challenges to hypertension management and healthy aging in rural China.

Beyond physical health, mental health has become an increasingly important public health concern. The WHO has identified the mental health of older adults as a key global public health issue [8]. Approximately 1.17 billion people worldwide were living with mental disorders in 2023, representing a 24.2% increase in the age-standardized prevalence rate between 1990 and 2023 [9]. Substantial evidence suggests that hypertension and mental health problems are bidirectionally associated, whereby psychological distress, depression, and anxiety can elevate blood pressure through neuroendocrine and behavioral mechanisms, while long-term hypertension can in turn increase psychological distress [10,11]. These comorbid mental health conditions may significantly exacerbate the clinical progression of hypertension in older adults, predisposing them to heightened susceptibility to severe hypertension-related complications. Coexisting psychological distress may also complicate medication adherence and other aspects of disease self-management [12]. Therefore, prioritizing the mental health of older adults with hypertension should be a vital focus for comprehensive healthcare initiatives and community-based interventions.

Negative life events, such as bereavement, serious illness, financial difficulties, and family conflicts, are important psychosocial stressors in later life. Studies conducted among older adults in Singapore, Brazil, and China have linked negative life events to more depressive symptoms or poorer psychological well-being across different sociocultural settings [13,14,15,16,17]. The number of negative life events has also shown a dose–response association with depressive symptoms, with a greater burden of depressive symptoms observed among rural older adults [16]. Beyond psychological well-being, negative life events have been associated with cognitive functioning in later life. The death of a child or grandchild has been associated with faster cognitive decline, although these associations vary across different types of life events [18]. Stressful life events may also be relevant to Alzheimer’s disease, but current evidence remains insufficient to establish whether they contribute to disease onset or progression [19]. However, how individual psychological and social resources jointly relate to this association remains insufficiently understood, particularly among rural older adults with hypertension.

According to Bandura’s Self-Efficacy Theory, self-efficacy refers to individuals’ beliefs in their ability to organize and execute the actions required to manage challenging situations [20]. Negative life events may undermine individuals’ perceived control and confidence in coping with difficulties, whereas higher self-efficacy may help individuals maintain confidence and adopt more constructive coping strategies when facing stress [20,21]. Previous studies have shown that individuals with higher self-efficacy scores tended to experience fewer depressive symptoms [12,22,23,24], and negative life events are associated with significantly lower levels of self-efficacy [21,25]. Thus, self-efficacy may be particularly relevant to older adults with hypertension, who face ongoing demands related to medication adherence, blood pressure monitoring, and lifestyle management. Among patients with hypertension, self-efficacy has been associated with depressive symptoms and self-care behaviors [12], while higher self-efficacy has generally been linked to better disease self-management [26].

Social capital refers to resources accessible through social relationships, including emotional support, practical assistance, and information [27,28]. According to the stress-buffering framework, accessible social resources may reduce the psychological burden associated with stressful experiences by providing support and assistance when individuals encounter difficulties [29,30,31,32,33,34]. Empirical evidence among Chinese older adults supports this perspective. Friendship ties have been found to moderate the association between negative life events and depressive symptoms, particularly among rural and socioeconomically disadvantaged older adults [17], while family support has also shown a significant moderating role in other research [35].

Beyond this direct association, social capital may also modify the relationship between self-efficacy and psychological distress [33,34]. When social resources are limited, individuals may rely more heavily on their own confidence and problem-solving abilities to cope with difficulties. In contrast, access to interpersonal support and practical assistance may make individuals less dependent on their personal coping confidence. Previous research among rural older adults with hypertension has also identified relationships among social capital, self-efficacy, and quality of life [36]. However, whether social capital moderates the association between self-efficacy and psychological distress remains unclear. Furthermore, the Resource Generator China scale (RG-China) measures individual social capital in terms of access to resources for daily living, expert advice, personal skills, and problem-solving, providing a resource-based perspective that is particularly relevant to rural older adults [28].

**Figure 1:**
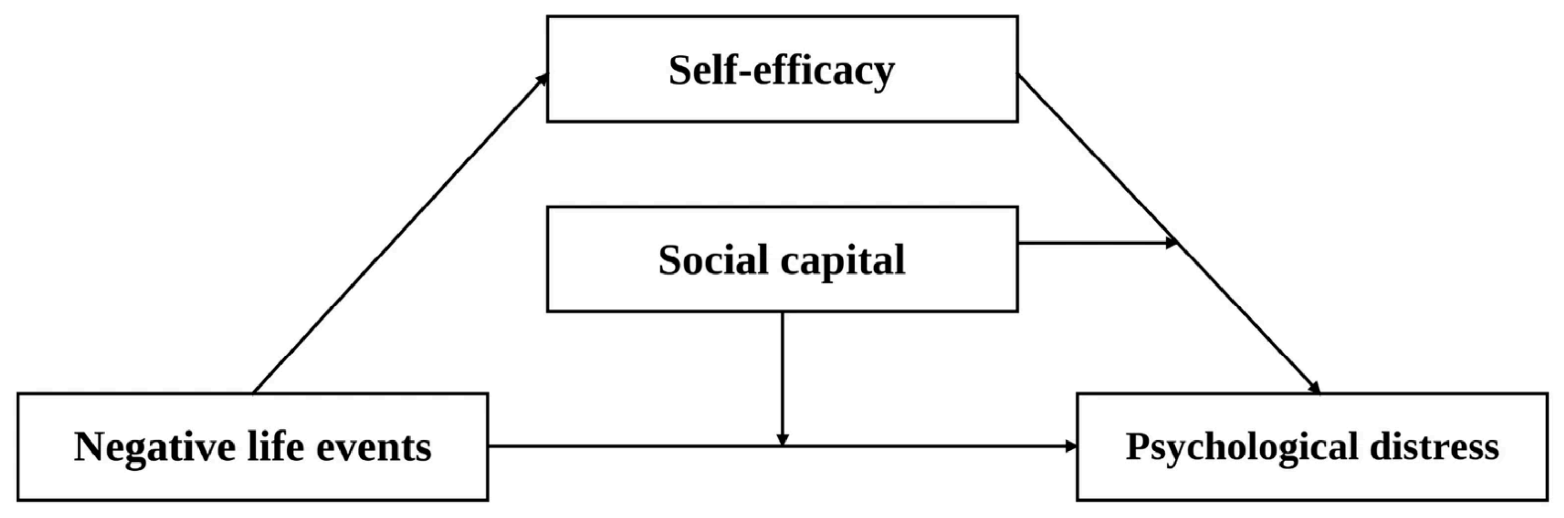
Theoretical model: a moderated mediation model.

To address these research gaps, this study examined the associations among negative life events, self-efficacy, social capital, and psychological distress among rural older adults with hypertension. Specifically, we hypothesized that (1) negative life events would be positively associated with psychological distress; (2) self-efficacy would mediate the relation between negative life events and psychological distress; and (3) social capital would moderate the direct path in the association between negative life events and psychological distress, and moderate the latter half of the mediation path in the association between negative life events and psychological distress. The research hypotheses were examined by a moderated mediation model (Figure 1).

## 2. Materials and Methods

### 2.1. Participants

This cross-sectional study was conducted in rural Shandong Province, China, from June to July 2021. A multistage stratified random sampling framework was employed. Based on socioeconomic development and geographical location, three study areas were selected: Rushan (eastern Shandong, relatively high economic development), Laiwu (central Shandong, intermediate economic development), and Shanxian (western Shandong, relatively low economic development). Within each study area, three townships were randomly selected, followed by four villages within each township. Using local hypertension registries as the sampling frames, 20–30 older adults with previously diagnosed hypertension were randomly selected from each village.

The sample size was estimated using a previously reported hypertension prevalence of 53% [37], a 95% confidence level, and an absolute precision of 0.032, yielding a minimum required sample size of approximately 935. Considering potential nonparticipation, the target sample size was set at 960 [38].

The inclusion criteria were as follows: (1) aged 60 years or older; (2) previously diagnosed with hypertension; (3) able to communicate and participate in the interview; and (4) willing to participate in the survey. Individuals who did not meet these criteria were excluded.

Before data collection, all investigators received standardized training. With the assistance of local healthcare providers and community workers, trained investigators conducted structured face-to-face interviews at village clinics or village committee offices. Completed questionnaires were reviewed by quality supervisors to ensure data quality. A total of 965 older adults were approached, all of whom participated in the survey (response rate: 100%). After excluding 12 participants who did not meet the study-specific eligibility criteria, 953 participants (98.8%) were included in the final analysis.

Informed consent was obtained from all participants before the survey.

### 2.2. Measures

#### 2.2.1. Negative Life Events

In this study, the negative life events exposure was assessed by the self-reported experience of a negative life event within the past 12 months [15]. Seven types of events were considered: (1) serious diseases (self or family member); (2) financial crises; (3) the death of a family member or a loved one (spouse, children, or relatives); (4) serious financial loss; (5) natural calamities; (6) experiencing problems in a relationship with family members, relatives, or close friends; and (7) experiencing accident or other frightening experiences. The number of negative life events within the past 12 months were counted and further categorized (“0” = 0, “1” = 1, “≥2” = 2), consistent with previous research among Chinese older adults [16]. This categorization distinguished participants who experienced no events, a single event, or multiple events, allowing the cumulative occurrence of negative life events to be examined. Each event was counted equally, regardless of its type. The scale has been widely used in Chinese older-adult studies and demonstrated good internal consistency [15].

#### 2.2.2. Psychological Distress

Psychological distress was assessed using the validated Chinese version of 10-item Kessler Psychological Distress Scale [39]. The K10 consists of 10 items assessing symptoms of psychological distress experienced during the preceding four weeks. Each item is rated on a five-point scale according to symptom frequency, with higher scores indicating more frequent symptoms. The response options range from 1 to 5 according to the frequency in the past four weeks with 1 meaning almost never, 2 meaning occasionally, 3 meaning some of the time, 4 meaning most of the time, and 5 meaning all of the time. The K10 total score ranges 10 to 50 points, with higher scores indicating greater psychological distress. In this study, Cronbach’s α coefficient of the K10 was 0.893.

#### 2.2.3. Self-Efficacy

Self-efficacy was measured using the General Self-Efficacy Scale (GSES) developed by Schwarzer and Jerusalem [40,41]. This instrument was selected because it measures individuals’ general confidence in coping with difficulties and stressful situations, which is directly relevant to the hypothesized relationship between negative life events and psychological distress. Unlike disease-specific self-efficacy instruments, the GSES captures coping confidence across different life situations. The GSES consists of 10 items rated on a 4-point Likert scale (from 1 = “not at all true” to 4 = “exactly true”), with total scores ranging from 10 to 40, and higher scores indicating greater self-efficacy [41]. The Chinese version of the GSES has been validated and widely used among older adults and individuals with chronic diseases in China [42]. In the present study, the scale showed excellent reliability (Cronbach’s α = 0.93).

#### 2.2.4. Social Capital

The Resource Generator Scale for China (RG-China) was used to assess individual social capital among rural older adults with hypertension. The scale was adapted and validated among rural Chinese older adults, with previous studies supporting its reliability and construct validity [28,38,43]. RG-China has four dimensions: domestic resources (eight items), expert advice (six items), personal skills (six items) and problem-solving resources (five items). Domestic resources refer to resources that can help or improve the quality of daily life; expert advice refers to resources that are helpful for medical treatment, financial management, and agricultural production; personal skills refer to the resources that provide domestic maintenance assistance and enrich entertainment activities; and problem-solving resources refer to resources that help solve personal or family problems and provide moral or material support. The RG-China consists of 25 items, each with a binary response option (yes = 1; no = 0). Total and subscale scores were calculated by summing the corresponding item scores, with higher scores indicating greater access to social capital resources. Cronbach’s α was 0.836 in the present sample.

#### 2.2.5. Control Variables

The control variables in this study included gender, age (≤70; >70), marital status (married; single including never married, divorced, and widowed), education (middle school and above; primary school; illiteracy), household income per year (<10,000 Chinese yuan; 10,000~30,000 Chinese yuan; ≥30,000 Chinese yuan), living arrangement (living with others; living alone), cigarette smoking (no; yes), alcohol drinking (no; yes), physical exercise (occasional; regular; always), control of blood pressure (no; yes), and multimorbidity (no; yes). In this study, illiteracy was defined as having never attended school. For descriptive comparisons, age was categorized as ≤70 and >70 years, consistent with previous research among rural older adults with hypertension and the age distribution of our sample [44].

Cigarette smoking was assessed by asking participants, “Do you currently smoke (within the past six months)?” (yes/no). Alcohol drinking was originally classified into five categories: never drinking, former drinking, rarely drinking, drinking sometimes, and frequent drinking. Never and former drinkers were categorized as non-drinkers, while the remaining categories were classified as drinkers. Physical exercise was assessed based on exercise frequency during the preceding 12 months, excluding occupational physical activity. Six original response categories were regrouped into occasional (never or almost never, less than once per month, or 1–3 times per month), regular (1–2 or 3–4 times per week), and always (at least five times per week).

Multimorbidity was defined as the presence of at least one additional chronic condition beyond hypertension, based on participants’ reported diagnoses and supporting diagnostic information. Blood pressure was measured twice, at the beginning and end of the interview, approximately 30 min apart. Control of blood pressure was defined as a mean systolic blood pressure <140 mmHg and a mean diastolic blood pressure <90 mmHg.

Covariates were selected a priori based on previous epidemiological studies of psychological distress and hypertension among older adults, taking into account sociodemographic characteristics, health-related behaviors, and health conditions [12,14,15,16,17,20,21,22,23,25,29,30,31,45].

### 2.3. Data Analysis

We used IBM SPSS Statistics, version 25.0 (IBM Corp., Armonk, NY, USA), and the PROCESS macro for SPSS, version 3.5 (developed by Andrew F. Hayes), to analyze the data. Means and standard deviations were calculated for continuous variables, and frequencies and percentages for categorical variables. The normality of K10 score distributions was assessed before group comparisons. Independent-samples *t*-tests and one-way analysis of variance (ANOVA) were used for comparisons between two and three or more groups, respectively, when the relevant parametric assumptions were satisfied. Significant omnibus ANOVA results were further examined using post hoc pairwise comparisons with adjustment for multiple testing. Spearman’s rank correlation coefficients were used to examine associations among negative life events, self-efficacy, social capital, and psychological distress, given the discrete and ordinal nature of negative life event exposure.

Multivariable linear regression analyses were performed to examine the hypothesized associations while adjusting for potential confounders. Path analyses were conducted to examine the association between negative life events and psychological distress and the mediating role of self-efficacy. PROCESS Model 4 was first used to estimate the total, direct, and indirect associations. The mediation model was specified as follows:

$$Y=\alpha_{1}+cX+\gamma_{1}^{T}C+\varepsilon_{1}$$

$$M=\alpha_{2}+aX+\gamma_{2}^{T}C+\varepsilon_{2}$$

$$Y=\alpha_{3}+c^{\prime }X+bM+\gamma_{3}^{T}C+\varepsilon_{3}$$

Here, X represents negative life events, M represents self-efficacy, Y represents psychological distress, and C represents the covariates included in the regression equations. The coefficient a represents the association between negative life events and self-efficacy, b represents the association between self-efficacy and psychological distress after adjustment for negative life events, c represents the total association, and $c^{\prime }$ represents the direct association. The indirect association was quantified as ab=a×b, with$\;c=c^{\prime }+ab$.

Additional regression analyses were conducted to examine whether social capital moderated the associations of negative life events with psychological distress and self-efficacy. Subsequently, PROCESS Model 15 was used to examine the moderating role of social capital in the direct pathway and the second stage of the indirect pathway. The moderated mediation model was specified as follows:

$$M=\alpha_{4}+aX+\gamma_{4}^{T}C+\varepsilon_{4}$$

$$Y=\alpha_{5}+c^{\prime }X+bM+qW+d\left(X\times W\right)+e\left(M\times W\right)+\gamma_{5}^{T}C+\varepsilon_{5}$$

Here, W represents social capital. Variables involved in interaction terms were mean-centered before constructing the product terms. The interaction term X×W examines whether social capital moderates the association between negative life events and psychological distress, whereas M×W examines whether social capital moderates the association between self-efficacy and psychological distress. The conditional direct and indirect associations were expressed as$\;\mathrm{Direct}\left(w\right)=c^{\prime }+dw$ and$\;\mathrm{Indirect}\left(w\right)=a\left(b+ew\right)$, respectively, where w represents a specified level of social capital. These associations were estimated at three representative levels of social capital (mean − 1SD, mean, and mean + 1SD).

Categorical covariates were numerically coded and entered as adjustment variables. Continuous variables were standardized before regression analysis, and variables involved in interaction terms were mean-centered before constructing the product terms. Bootstrap confidence intervals were estimated using 5000 resamples. Indirect associations were considered statistically significant when their 95% confidence intervals excluded zero. Interaction terms were evaluated using their regression coefficients and corresponding significance tests. Regression assumptions were assessed through residual diagnostics, including examinations of linearity, residual normality, and homoscedasticity. No substantial violations were identified in these assessments. Multicollinearity was evaluated using variance inflation factors (VIFs), all of which were below 5. Statistical significance was defined as a two-sided *p*-value < 0.05.

## 3. Results

### 3.1. Descriptive Analyses

The study included 953 participants, and their characteristics are presented in Table 1. The average age in the sample was 70.50 ± 6.11 years old, with 61.4% of them being female. Education varied in the sample, with more than half of the participants having less than a middle school education (73.3%). Regarding annual household income, 11.4% reported an income level of more than 30,000 Chinese yuan, while 56.8% reported an income level of less than 10,000 Chinese yuan. Among the participants, 72.4% were nonsmokers, 67.1% did not consume alcohol, and 63.3% reported exercising at least once per week. Furthermore, 68.5% had uncontrolled blood pressure, and 73.1% had multimorbidity. Univariate analyses revealed significant differences in psychological distress scores according to gender, marital status, education, household income, living arrangements, cigarette smoking, alcohol drinking, physical exercise, and multimorbidity (*p* < 0.05).

### 3.2. Correlation Between Key Study Variables

Table 2 presents the means, standard deviations, and correlation matrix for the key study variables. Negative life events were positively correlated with psychological distress (r = 0.432) and negatively correlated with self-efficacy (r = −0.238) and social capital (r = −0.169). Self-efficacy was positively correlated with social capital (r = 0.156) and negatively correlated with psychological distress (r = −0.449). Social capital was also negatively correlated with psychological distress (r = −0.255) (all *p* < 0.01).

### 3.3. Mediation Effect Analysis

Table 3 and Table 4 present the results of the mediation analysis. Negative life events were positively associated with psychological distress (total association: β = 0.418, t = 15.507, *p* < 0.001) and negatively associated with self-efficacy (β = −0.199, t = −6.529, *p* < 0.001). After accounting for negative life events, self-efficacy was negatively associated with psychological distress (β = −0.265, t = −9.642, *p* < 0.001), while the coefficient for negative life events was reduced to 0.365 (t = 13.884, *p* < 0.001). The indirect association through self-efficacy was 0.053 (95% CI: 0.035–0.073), accounting for 12.70% of the total association.

### 3.4. Moderated Mediation Analysis

The results of the moderated mediation analysis are presented in Table 5 and Table 6. The interaction between negative life events and social capital was significantly associated with psychological distress (β = −0.058, t = −2.135, *p* < 0.05), as was the interaction between self-efficacy and social capital (β = 0.059, t = 2.227, *p* < 0.05). These findings indicate that the positive association between negative life events and psychological distress was weaker at higher levels of social capital, whereas the negative association between self-efficacy and psychological distress became less pronounced. Conditional direct associations were 0.382, 0.326, and 0.271, and conditional indirect associations were 0.060, 0.048, and 0.037 at low, mean, and high levels of social capital, respectively. Furthermore, the index of moderated mediation was −0.012 (BootSE = 0.006, 95% CI: −0.023 to −0.001), indicating that the indirect association between negative life events and psychological distress through self-efficacy varied significantly across levels of social capital.

**Figure 2:**
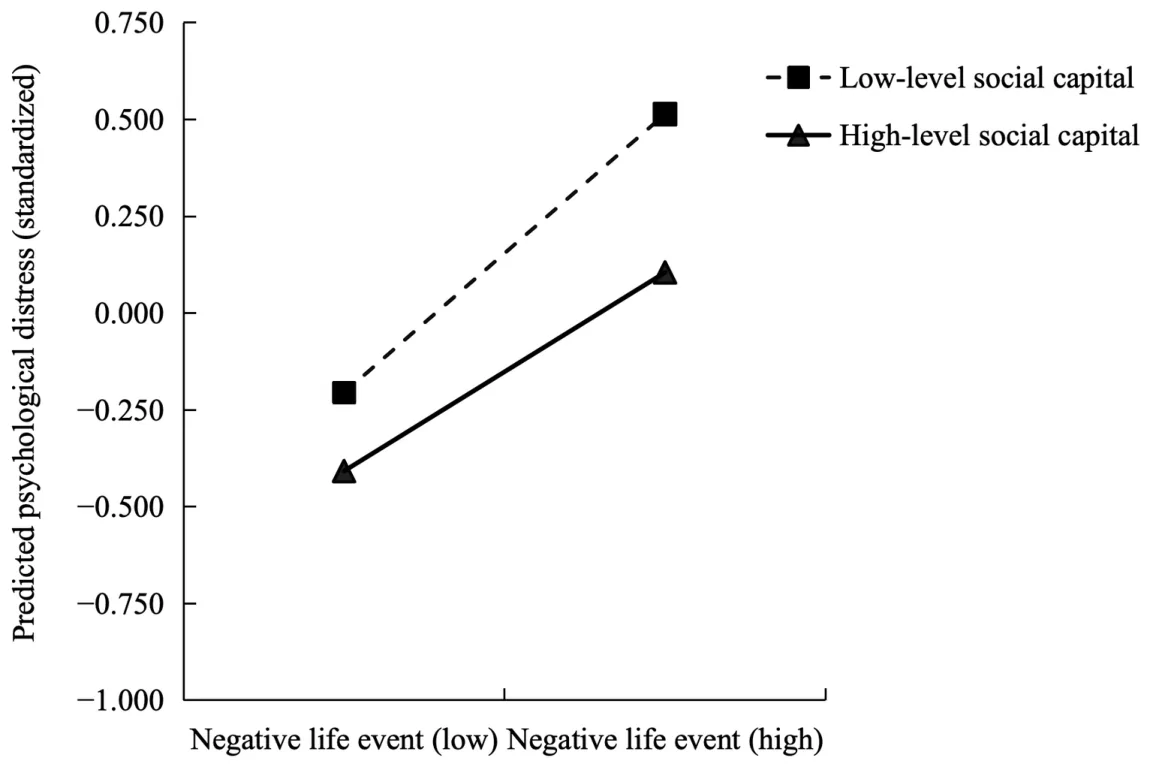
Moderating effect of social capital on the association between negative life events and psychological distress.

**Figure 3:**
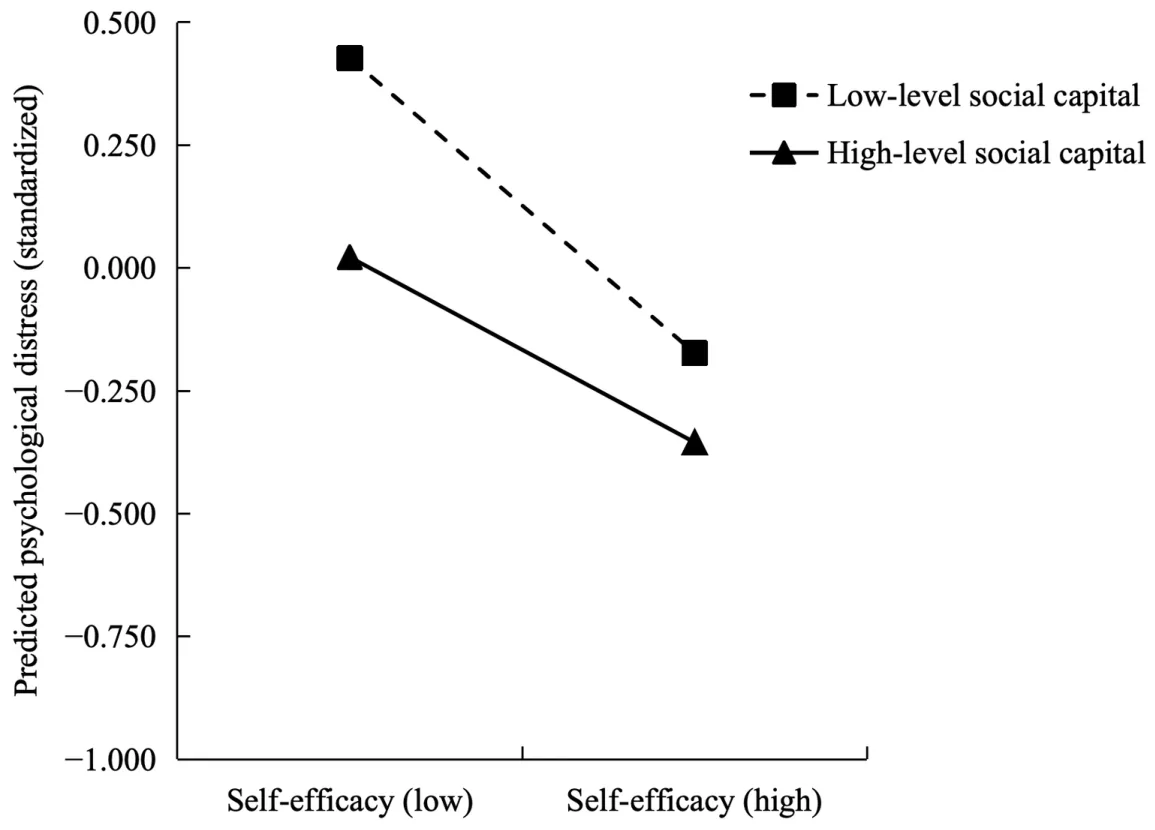
Moderating effect of social capital on the association between self-efficacy and psychological distress.

The moderating effect can be visualized in Figure 2 and Figure 3. To illustrate the moderating effect, social capital was divided into high-level and low-level groups based on scores higher or lower than one standard deviation. As shown in Figure 2 and Table 6, negative life events were positively associated with psychological distress at both lower (β = 0.382, bootstrapped SE = 0.034, 95% CI [0.315, 0.448]) and higher levels of social capital (β = 0.271, bootstrapped SE = 0.040, 95% CI [0.193, 0.350]). The association was stronger at lower levels of social capital, indicating that social capital weakened the positive association between negative life events and psychological distress. As illustrated in Figure 3, greater self-efficacy was associated with lower predicted psychological distress at both levels of social capital, and this inverse association was stronger at lower levels of social capital, consistent with the significant interaction between self-efficacy and social capital (β = 0.059, t = 2.227, *p* < 0.05). The conditional indirect effect of negative life events on psychological distress through self-efficacy was 0.060 (bootstrapped SE = 0.012, 95% CI [0.038, 0.085]) at one standard deviation below the mean of social capital and 0.037 (bootstrapped SE = 0.009, 95% CI [0.020, 0.057]) at one standard deviation above the mean. Thus, the indirect association through self-efficacy was weaker at higher levels of social capital.

## 4. Discussion

In this study, negative life events were positively associated with psychological distress among rural older adults with hypertension, with self-efficacy accounting for part of this association. Social capital moderated both the direct association between negative life events and psychological distress and the indirect association through self-efficacy. These findings shed light on the potential factors influencing psychological distress and provide insights for developing targeted interventions to improve mental health in older rural adults with hypertension. In addition, to our knowledge, this is the first study to explore the association between negative life events and psychological distress among older adults with hypertension, as well as the mediating role of self-efficacy and the moderating role of social capital.

The positive association between negative life events and psychological distress is consistent with previous evidence linking adverse life experiences to depressive symptoms among older adults [16,17,46]. Both the types and cumulative number of negative life events have been associated with depressive symptoms in later life [47,48,49]. As individuals age, they may increasingly encounter stressful experiences, including bereavement, family disruptions, serious illness, financial difficulties, and declining physical functioning. Such experiences may be particularly challenging for older adults with hypertension, who must also manage the ongoing demands of chronic illness. The cognitive vulnerability–stress framework provides a possible explanation for this association [50,51]. Negative life events may trigger sadness, anxiety, uncertainty, or feelings of helplessness, while cognitive vulnerabilities, such as negative attributional styles and pessimistic interpretations of stressful experiences, may prolong these emotional responses and contribute to psychological distress [52]. The accumulation of adverse experiences, together with the psychological demands of managing hypertension, may therefore help explain the greater psychological distress observed among participants reporting more negative life events.

Our mediation analyses further revealed a significant mediating effect of self-efficacy on the association between negative life events and psychological distress. Self-efficacy may be particularly relevant to how older adults respond to these challenges. Individuals with higher self-efficacy tend to have greater confidence in their ability to manage difficult situations, engage in active problem-solving, and adopt constructive coping strategies rather than relying on avoidance or other maladaptive responses [20,24]. Conversely, lower self-efficacy may be accompanied by greater perceived helplessness and difficulty managing stressful circumstances. For older adults with hypertension, confidence in managing medication, maintaining healthy behaviors, and addressing disease-related difficulties is especially important, as self-efficacy has been consistently associated with hypertension self-care behaviors [26]. When confronted with negative life events, older adults with greater self-efficacy may be better positioned to maintain a sense of control over daily life and disease management, which could be relevant to their psychological well-being.

Social capital was negatively associated with psychological distress in our study, consistent with previous findings [53,54,55,56]. Social capital may be particularly important for rural older adults with hypertension, as support from family members, relatives, friends, and neighbors can provide emotional reassurance, practical assistance, and information about disease treatment, healthcare services, and relevant policies [28,57]. These resources may help older adults manage everyday difficulties, maintain a positive outlook, and remain socially engaged. Evidence from rural China has shown that older adults with more diverse social networks reported fewer depressive symptoms, whereas those with restricted social networks experienced greater psychological difficulties [58]. Intergenerational emotional support has also been associated with fewer depressive symptoms, while the association with financial support varies according to living arrangements and chronic disease burden [59]. Beyond the availability of social contacts, social isolation and loneliness represent important dimensions of social relationships in later life. Social isolation generally refers to limited social contact, whereas loneliness reflects the subjective perception that one’s social relationships are insufficient or unsatisfactory [60]. Longitudinal evidence further indicates that social disconnectedness is associated with subsequent depressive and anxiety symptoms, partly through perceived social isolation [61]. These findings highlight the importance of both accessible social resources and the quality of interpersonal relationships for psychological well-being in later life.

The positive association between negative life events and psychological distress was weaker at higher levels of social capital. This pattern is consistent with the stress-buffering framework, which emphasizes the importance of accessible social resources when individuals encounter stressful circumstances [33,34]. When faced with bereavement, serious illness, financial difficulties, or family conflicts, older adults with greater social capital may be more likely to receive emotional comfort, practical assistance, and useful advice. Emotional support may help them manage distressing experiences, while practical assistance and access to relevant information may make everyday challenges more manageable [33,57]. Previous research among Chinese older adults has shown that social support can moderate the association between stressful life events and depressive symptoms, although the roles of different social relationships vary. One study identified a significant moderating role for family support [35]. In contrast, a longitudinal study found that friendship ties significantly moderated the association between negative life events and depressive symptoms, particularly among rural and socioeconomically disadvantaged older adults. However, no statistically significant moderating association was observed for family ties in that study [17]. Research comparing urban and rural Chinese older adults has further highlighted the relevance of family connectivity and perceived filial support to depressive symptoms in rural settings [62]. These findings suggest that family members, friends, and neighbors may provide distinct but complementary forms of assistance. For rural older adults with hypertension, family support may be particularly relevant to daily care and disease management, while friendships and neighborhood relationships may provide companionship, opportunities for social participation, and additional support during stressful experiences [58,62].

The negative association between self-efficacy and psychological distress was also weaker at higher levels of social capital, indicating that self-efficacy was more strongly associated with lower psychological distress among older adults with limited social resources. One possible explanation is that individual psychological resources and accessible social resources may play partly compensatory roles when older adults encounter stressful circumstances. Individuals with limited social resources may depend more heavily on their own confidence, problem-solving abilities, and self-management skills to cope with difficulties [20,26]. In contrast, older adults with greater access to interpersonal assistance, health-related information, and emotional support may be less dependent on individual coping confidence when facing similar challenges. Previous research among rural empty-nest older adults with hypertension in Shandong examined the mediating role of self-efficacy in the association between social capital and quality of life [36]. Social capital and self-efficacy may be closely connected in the daily management of hypertension, but they represent different resources. Social capital provides access to information, assistance, and interpersonal support, whereas self-efficacy reflects individuals’ confidence in their ability to manage health-related tasks and respond to difficulties. For example, assistance from family members or neighbors may help older adults obtain treatment information, attend medical appointments, or manage daily activities. Such assistance may make difficult situations more manageable even when personal confidence is relatively low. Conversely, when external assistance is limited, individuals may need to rely more heavily on their own confidence and problem-solving abilities to manage similar challenges.

Stressful life events in later life may also be relevant to cognitive functioning and the ability to manage everyday responsibilities. Longitudinal research has shown that different types of negative life events are associated with different patterns of cognitive change, with the loss of a child or grandchild associated with a faster rate of cognitive decline among older adults [18]. Social resources may also be relevant to cognitive health. A longitudinal study of Chinese older adults found that financial support and reciprocity were associated with better subsequent executive functioning, while social participation was associated with better episodic memory [63]. Cognitive functioning is particularly important when older adults must make decisions about medical treatment, financial matters, and daily care arrangements. Research involving healthy older adults and patients with Alzheimer’s disease found that financial capacity was associated with cognitive performance and diagnostic status, but not independently with recent stressful life events [64]. Related research has emphasized the importance of financial decision-making capacity in understanding older adults’ vulnerability to financial exploitation and the associated legal implications [65]. For rural older adults with hypertension, stressful experiences accompanied by cognitive or functional difficulties may create additional challenges in managing medical treatment, financial affairs, and everyday responsibilities, highlighting the importance of appropriate assessment and access to reliable family and community support.

These findings have practical implications for the provision of primary healthcare to rural older adults with hypertension. Community healthcare providers could incorporate brief assessments of recent negative life events, psychological distress, self-efficacy, and available social support into routine hypertension follow-up visits or home-based care. Particular attention could be given to older adults who have recently experienced bereavement, serious illness, or financial hardship, especially those reporting low self-efficacy or limited social resources. The WHO Integrated Care for Older People framework provides guidance for identifying psychological, functional, and social care needs and developing individualized care plans within primary and community healthcare settings [66]. For older adults experiencing difficulties with disease management, structured health education and individualized coaching could focus on setting achievable goals, medication management, blood pressure monitoring, and practical problem-solving skills. A randomized trial in rural China found that health coaching improved hypertension self-management and medication literacy, supporting the feasibility of this approach in rural healthcare settings [67]. For individuals with persistent or substantial psychological distress, primary healthcare providers could facilitate further assessment and referral to appropriate mental health services. Evidence from a randomized trial of collaborative care for depression in older primary care patients also supports coordinated management involving primary care professionals and mental health specialists [68].

Support beyond healthcare facilities may be equally important, particularly for rural older adults with limited social resources. Village healthcare workers and community organizations could help identify older adults who have difficulty obtaining assistance with daily activities, medical appointments, or disease-related decisions and connect them with available family, neighborhood, or community support. Regular home visits, peer-support activities, and opportunities for social participation could be considered according to individual preferences and local resources. Family members could also be encouraged to provide emotional support and participate in disease-management discussions when appropriate, while respecting older adults’ preferences and independence. A systematic review and meta-analysis found that interventions promoting social contact, developing interpersonal skills, and addressing social cognition were associated with reductions in loneliness among older adults [69]. These approaches provide potential directions for community-based support, particularly for individuals with restricted social networks. Future intervention studies could evaluate whether combining psychological assessment, self-management support, and assistance in accessing social resources is associated with improvements in psychological distress and quality of life among rural older adults with hypertension.

This study used the Resource Generator Scale for China to assess individual social capital and extended previous research on the associations among negative life events, self-efficacy, social capital, and psychological distress in rural older adults with hypertension. However, several limitations should be acknowledged. First, the cross-sectional design precludes causal inference, and the mediation and moderation findings should be interpreted as statistical associations. Second, self-reported measures may introduce recall, reporting, and common-method biases, potentially affecting the observed associations. Negative life events were categorized as none, one, or two or more events, without considering their severity or timing, which may obscure differences across experiences. Third, nonparticipation and the exclusion of individuals unable to communicate adequately may introduce selection bias, while unmeasured factors may contribute to residual confounding. In addition, the analyses did not account for within-village correlation arising from the multistage sampling design, potentially affecting the precision of the estimates. Finally, the study was conducted among rural older adults with hypertension in three counties of Shandong Province, limiting generalizability to other populations and settings. Future studies should adopt longitudinal and multicenter designs, incorporate more detailed assessments of negative life events and appropriate cluster-adjusted analyses, and evaluate targeted interventions integrating psychological support, hypertension self-management, and community resources.

## 5. Conclusions

In conclusion, negative life events were positively associated with psychological distress among rural older adults with hypertension, with self-efficacy accounting for part of this association. Social capital moderated both the direct and indirect associations, with higher levels of social capital associated with weaker positive associations between negative life events and psychological distress and weaker negative associations between self-efficacy and psychological distress. These findings highlight the relevance of individual psychological resources and social resources to psychological distress in this population. Incorporating psychological assessment, self-management support, and access to community resources into hypertension care may be worth exploring, although longitudinal and intervention studies are needed to evaluate their potential benefits.

## Figures and Tables

**Table 1:** Descriptive and univariate analysis of psychological distress among older adults with hypertension in Shandong, China, 2021 (*N* = 953).

Characteristics	*N* (%)	Psychological Distress	*t*/*F*	*p*-Value
**Gender**			8.069	<0.001
Male	368 (38.6)	15.58 ± 6.64		
Female	585 (61.4)	19.63 ± 8.06		
**Age**			0.663	0.507
≤70	453 (47.5)	18.25 ± 7.94		
>70	500 (52.5)	17.91 ± 7.66		
**Marital status**			4.301	<0.001
Single	195 (20.5)	20.19 ± 8.53		
Married	758 (79.5)	17.52 ± 7.50		
**Education**			25.298	<0.001
Illiterate	353 (37.0)	20.11 ± 8.23		
Primary school	346 (36.3)	17.71 ± 7.48		
Middle school or above	254 (26.7)	15.72 ± 6.81		
**Household income**			16.840	<0.001
<10,000 Chinese yuan	541 (56.8)	19.19 ± 7.98		
10,000~30,000 Chinese yuan	303 (31.8)	17.20 ± 7.63		
≥30,000 Chinese yuan	109 (11.4)	14.94 ± 5.97		
**Living arrangement**			−3.811	<0.001
Living with others	790 (82.9)	17.64 ± 7.54		
Living alone	163 (17.1)	20.17 ± 8.61		
**Cigarette smoking**			5.655	<0.001
No	690 (72.4)	18.94 ± 7.96		
Yes	263 (27.6)	15.79 ± 6.85		
**Alcohol drinking**			5.522	<0.001
No	639 (67.1)	19.03 ± 7.97		
Yes	314 (32.9)	16.11 ± 7.03		
**Physical exercise**			10.337	<0.001
Occasional	349 (36.6)	19.45 ± 8.38		
Regular	209 (21.9)	18.02 ± 7.23		
Always	395 (41.4)	16.87 ± 7.34		
**Control of blood pressure**			0.920	0.358
No	653 (68.5)	18.23 ± 7.75		
Yes	300 (31.5)	17.73 ± 7.87		
**Multimorbidity**			−7.495	<0.001
No	256 (26.9)	15.04 ± 6.06		
Yes	697 (73.1)	19.18 ± 8.06		

Note: Post hoc comparisons indicated significant pairwise differences across all educational and household-income categories (adjusted *p* < 0.05). For physical exercise, only the difference between the occasional and always groups was statistically significant (adjusted *p* < 0.001).

**Table 2:** Correlation analysis results between key study variables (*N* = 953).

Variables	Mean(SD)	1	2	3	4
Negative life event	1.07 ± 1.24	1			
2. Self-efficacy	25.12 ± 7.87	−0.238 **	1		
3. Social capital	18.65 ± 4.20	−0.169 **	0.156 **	1	
4. Psychological distress	18.07 ± 7.79	0.432 **	−0.449 **	−0.255 **	1

** *p* < 0.01.

**Table 3:** Mediation analysis of self-efficacy.

Regression Equation		Goodness of Fit		Coefficient Significance	
Outcome Variables	Process	*R* ^2^	*F* Value	*β* Value	*t* Value
Psychological distress	Negative life events	0.343	240.459	0.418	15.507 ***
Self-efficacy	Negative life events	0.160	42.631	−0.199	−6.529 ***
Psychological distress	Negative life events	0.402	178.450	0.365	13.884 ***
	Self-efficacy			−0.265	−9.642 ***

*** *p* < 0.001. Note: Standardized variables were substituted into the regression equation.

**Table 4:** Total, direct, and indirect associations between negative life events and psychological distress.

	Effect Size	SE	95% CI	Relative Effect Value
Total effect	0.418	0.027	(0.365, 0.471)	
Direct effect	0.365	0.026	(0.314, 0.417)	87.30%
Indirect effect	0.053	0.010	(0.035, 0.073)	12.70%

Note: Standardized variables were substituted into the regression equation. SE, standard error; 95% CI, 95% confidence interval.

**Table 5:** Regression results of the moderated mediation model.

Regression Equation		Goodness of Fit		Coefficient Significance	
Outcome Variables	Predictive Variables	*R* ^2^	*F* Value	*β* Value	*t* Value
Psychological distress		0.378	47.582 ***		
	Negative life events			0.372	13.715 ***
	Social capital			−0.165	−6.045 ***
	Negative life events × social capital			−0.089	−3.343 **
Self-efficacy		0.166	15.571 ***		
	Negative life events			−0.179	−5.694 ***
	Social capital			0.065	2.037 *
	Negative life events × social capital			0.045	1.449
Psychological distress		0.433	51.122 ***		
	Negative life events			0.326	12.372 ***
	Social capital			−0.147	−5.597 ***
	Negative life events × social capital			−0.058	−2.135 *
	Self-efficacy			−0.243	−8.988 ***
	Self-efficacy × social capital			0.059	2.227 *

*** *p* < 0.001, ** *p* < 0.01, * *p* < 0.05. Note: Standardized variables were substituted into the regression equation.

**Table 6:** Conditional direct and indirect associations and the index of moderated mediation.

	Social Capital	Effect Size	Bootstrapped SE	Bootstrapped *LLCI*	Bootstrapped *ULCI*
Direct effect	M − SD	0.382	0.034	0.315	0.448
	M	0.326	0.026	0.275	0.378
	M + SD	0.271	0.040	0.193	0.350
Indirect effect	M − SD	0.060	0.012	0.038	0.085
	M	0.048	0.009	0.032	0.067
	M + SD	0.037	0.009	0.020	0.057
Index of moderated mediation	-	−0.012	0.006	−0.023	−0.001

Note: M, mean; SD, standard deviation; SE, standard error; LLCI, lower limit confidence interval; ULCI, upper limit confidence interval. The index of moderated mediation evaluates whether the indirect association varies across levels of social capital.

## Data Availability

The data presented in this study are available from the corresponding author upon reasonable request. The data are not publicly available because they contain potentially sensitive individual-level information, and the informed consent obtained from participants did not include permission for public sharing of individual-level data.

## Abbreviations

The following abbreviations are used in this manuscript:

K10	Kessler Psychological Distress Scale
GSES	General Self-Efficacy Scale
RG-China	Resource Generator Scale for China
ANOVA	One-way analysis of variance
M	Mean
SD	Standard deviation
SE	Standard Error
CI	Confidence interval
LLCI	Lower limit confidence interval
ULCI	Upper limit confidence interval
